# Psychological Impact of the Lockdown Due to the COVID-19 Pandemic in University Workers: Factors Related to Stress, Anxiety, and Depression

**DOI:** 10.3390/ijerph18084367

**Published:** 2021-04-20

**Authors:** Alejandro Salazar, Jenifer Palomo-Osuna, Helena de Sola, Jose A. Moral-Munoz, María Dueñas, Inmaculada Failde

**Affiliations:** 1 The Observatory of Pain, Department of Biomedicine, Biotechnology and Public Health, Faculty of Nursing and Physiotherapy, University of Cádiz, 11009 Cádiz, Spain; alejandro.salazar@uca.es (A.S.); helena.desola.p@gmail.com (H.d.S.); joseantonio.moral@uca.es (J.A.M.-M.); maria.duenasro@uca.es (M.D.); inmaculada.failde@uca.es (I.F.); 2 Biomedical Research and Innovation Institute of Cádiz (INiBICA), Research Unit, Puerta del Mar University Hospital, University of Cadiz, 11009 Cádiz, Spain; 3 Department of Statistics and Operational Research, Faculty of Sciences, University of Cádiz, 11510 Puerto Real, Spain; 4 Preventive Medicine and Public Health Area, Department of Biomedicine, Biotechnology and Public Health, Faculty of Nursing and Physiotherapy, University of Cádiz, 11009 Cádiz, Spain; 5 Department of Nursing and Physiotherapy, Faculty of Nursing and Physiotherapy, University of Cadiz, 11009 Cadiz, Spain

**Keywords:** COVID-19, depression, anxiety, stress, university workers, psychological impact, lockdown

## Abstract

This study aims to explore the psychological impact of the coronavirus disease (COVID-19)-related lockdown in university workers, and to analyse the factors related to their levels of stress, anxiety, and depression. A cross-sectional study was conducted between 8–22 April 2020, 3.5 weeks after the COVID-19-related lockdown in Spain. We collected sociodemographic and occupational data, in addition to housing, work and health conditions. Coping strategies (Brief COPE-28); level of anxiety, stress, and depression (Depression Anxiety Stress Scales DASS-21); perception of the disease (COVID-19) (Brief Illness Perception Questionnaire BIPQ); and perceived level of social support (Escala Multidimensional de Apoyo Social EMAS) were measured. Multiple linear regression models were fitted to explore the factors related to the level of anxiety, depression, and stress. The sample included 677 subjects. Higher scores in depression, anxiety, and stress occurred among females, younger subjects, administration and service workers; and subjects with a smaller home, as well as those with worse health status, worse quality of sleep, and dysfunctional coping strategies. The COVID-19-related lockdown had a great impact on the mental health of university workers. The participants with specific sociodemographic and occupational characteristics, clinical disorders, and dysfunctional coping strategies were more at risk.

## 1. Introduction

In March 2020, the coronavirus disease (COVID-19) was declared a pandemic by the World Health Organization (WHO) (Geneva, Switzerland) [1], and the world had to face a new and very complicated situation that entailed a challenge for society. Spain was among the five most affected countries, suffering more severe outbreaks and high case-fatality rates [2]. To control this situation, the government took unprecedented actions with several implications for citizens [3,4,5].

One of the measures adopted was the declaration of a state of alarm in the country and the consequent confinement of citizens [6]. From the point of view of public health, it was a necessary measure for the common well-being. However, this situation, together with the impact of the disease itself, had economic, social, physical, and mental consequences. It has been shown that the social isolation is related to a decline in health and increase of symptoms of anxiety, depression, and even risk of suicidal ideation [7,8,9]. In addition, the concern and fear of contagion, the lack of specific information about the situation, and the stigma caused by the disease, have also been related to higher stress levels and a decrease in the mental health of the population [10].

Previous studies conducted in China have shown that the COVID-19 outbreak has caused mental problems in the population [11,12]. In the same vein, other studies carried out in different countries, most of them in health care workers, have shown similar results [13,14,15,16,17]. 

Two interesting reviews [4,5] analyzing the psychological consequences derived from the COVID-19 pandemic and previous severe acute respiratory syndrome coronavirus 1 (SARS-CoV-1) have highlighted the presence of post-traumatic stress disorder, anxiety, depression, and stress in patients during the previous SARS-CoV-1 epidemic, and these conditions can be long-lasting. In addition, other authors have found that anxiety is the most common problem, and is frequently associated with impaired sleep [18,19]. 

Some other risk factors of depression, anxiety and stress during lockdowns have also been studied. The list includes sociodemographic-, psychological-, housing-, social-, and work-related factors, as well as current or previous medical conditions [4,20]. Women in particular have been shown to be more vulnerable to stress, anxiety, and depression in this context [21,22], while living alone also increases anxiety levels [23]. In addition, worse housing conditions, including small homes and a lack of open areas, were related to more severe depressive symptoms [24] and higher perceived stress load. Receiving less family support also increased the risk of psychological symptoms during the lockdown [25,26]. Finally, health-related factors, including poor self-rated health [27], poor sleep quality [26], the presence of COVID-19 symptoms [19], and having a high-risk of COVID-19 family member [15] have also been reported to be risk factors of depression, anxiety, and stress in lockdown situations. 

In view of the above, and given the scarce knowledge about the effect of lockdown on university workers, we proposed a study to determine the psychological impact of the lockdown due to the COVID-19 pandemic in this specific group of workers. Additionally, we aim to know the factors related to the level of stress, anxiety, and depression in this population.

## 2. Materials and Methods

### 2.1. Design, Population, and Sample

This is a cross-sectional study carried out in a population of university workers in southern Spain identified from the census and contacted via their institutional email. The census of the University of Cádiz in March 2020 for each of the two labour sectors (research and teaching personnel (RTP) and administration and services personnel (ASP)) included 2047 people in RTP and 842 in ASP. As we had the complete census, we tried to contact all of the workers, and no selection rules were applied. A total of 677 workers responded to the survey (23.43% response rate). The response rate was 20.66% in RTP and 30.17% in ASP.

### 2.2. Information Collection

The information collection took place between 8 April and 22 April 2020, after three and a half weeks of COVID-19-related lockdown. We used an online survey in Google Forms.

### 2.3. Instruments and Variables

The survey was structured in seven sections, including sociodemographic data; knowledge of COVID-19 disease; contact and health conditions related to COVID-19; and impact of the lockdown on their lives, on their job, and personal economy. Emotional aspects and attitudes towards the COVID-19 pandemic, as well as concerns arising from the COVID-19 pandemic were also collected. For the purposes of this study, we focused on the sociodemographic data, housing, labour sector (RTP and ASP), health status, previous chronic conditions, characteristics of sleep, and care tasks.

A number of validated scales were used. First, the Brief COPE-28 was used to measure coping strategies. It has been validated in Spanish by Morán et al. [28]. It consists of 28 items, which determine 14 subscales: active coping, planning, emotional support, instrumental support, religion, positive reframing, acceptance, denial, humour, self-distraction, self-blame, behavioral disengagement, venting, and substance use. The scores of each subscale range between 2 and 8 points, with higher scores indicating greater use of that coping strategy. The 14 subscales are also classified into three dimensions: emotion-focused strategies (scoring 10 to 40), problem-focused strategies (scoring 6 to 24), and dysfunctional coping strategies (scoring 12 to 48) [29]. The first dimension is composed of the subscales emotional support, positive reframing, acceptance, religion, and humour; the second one of active coping, planning, and instrumental support; and the third is composed of denial, self-distraction, self-blame, behavioral disengagement, venting, and substance use. A more in-depth description of the internal structure of the scale, with the definitions of each subscale, can be seen in Table S1. A higher score on these dimensions indicates a greater use of these strategies [29].

The level of anxiety, stress, and depression were measured using the Depression Anxiety Stress Scale-21 (DASS-21). It has shown good psychometric properties and has been validated in Spanish by Daza et al. [30]. It consists of 21 items that are grouped into three dimensions (depression, anxiety, and stress). Each dimension is scored from 0 to 42, where 42 is the worst state for that dimension.

The Brief Illness Perception Questionnaire (BIPQ), adapted and validated in Spanish [31], was used to assess the perception of the COVID-19. This instrument includes eight items that return an overall score between 0 and 80, where a higher score indicates a worse perception of the disease [32].

The level of social support perceived by the participants was measured using the Multidimensional Scale of Perceived Social Support (EMAS, for its acronym in Spanish). This scale has shown good psychometric properties and has been validated in Spanish by Landeta and Calvete [33]. It consist of 12 items, with an overall score that evaluates perceived social support in general, and three specific dimensions that measure perceived social support from friends, family, and a special person [34]. The global score ranges from 12 to 84 points, and each dimension from 4 to 28 points, with higher scores indicating greater perceived support [35].

### 2.4. Analyses

A descriptive analysis was performed, showing the absolute and relative frequencies for the qualitative variables, and the mean and the standard deviation for the quantitative variables. In the latter case, normality in distribution was checked using the Kolmogorov–Smirnov test.

The factors related to the level of anxiety, depression, and stress were first analysed via bivariate analyses, with the Spearman rank correlation coefficient in the case of quantitative factors, the Mann–Whitney test for dichotomous factors, and the Kruskal–Wallis test for factors with three or more categories. Additionally, three multiple linear regression models were fitted. The dependent variables were the level of anxiety, level of depression, and level of stress, respectively. The potential factors tested were those described in the instruments and variables section, according to their clinical relevance and statistical significance, based on the bivariate analyses. A stepwise regression method was used in all the cases, selecting the potential factors in each step according to the results of the Wald test. The goodness of fit was checked via the coefficient of determination (*R*-square). The significance level was set at 0.05 in all the cases. The estimations of the parameters are shown along with their 95% confidence interval. All the analyses were performed in SPSS v.24 (IBM Corp., Armonk, NY, USA).

### 2.5. Ethical Aspects

The email sent to the university workers included the objective of the study, the person responsible, and the research group conducting it. Their anonymous and voluntary participation was requested. The study was conducted in accordance with international ethical standards contained in the Declaration of Helsinki. All the content was confidential in accordance with national and international legislation regarding the protection of personal data and the guarantee of digital rights.

## 3. Results

### 3.1. Characteristics of the Sample

A total of 677 subjects were included in the sample, 50.1% of whom were women, and the average age was 48.75 (*SD* = 10.51). Most people were married or in a relationship (72.6%), and only 1% were widow(er)s. A total of 62.5% of the respondents were RTP. Most of the participants indicated that they were not living with children under 14 years old (73.5%) or with people dependent on their care (79.7%). Regarding home features, 73% lived in a house between 50 and 129 m^2^, and 58.6% had open areas such as a garden or patio (Table 1).

Most of the respondents reported having the same health status as before the state of alarm (80.5%). In addition, 33.1% had a personal history of chronic disease, the most frequent being hypertension (39.3%), respiratory disease (27.7%), and chronic pain (13.9%). In the case of chronic pain, 41% of the subjects reported this being worse or much worse than before the lockdown. Regarding sleep, 88.2% of the sample slept between 6 and 8 hours, 48.9% had trouble sleeping at night sometimes or usually, 42.8% had trouble falling asleep, and 23.8% dreamed about the lockdown (Table 1).

The most common coping strategies used by the participants were acceptance (mean = 6.46; *SD* = 1.11), active coping (mean = 5.63; *SD* = 1.36), and planning (mean = 5.39; SD = 1.46), and the least common were substance use (mean = 2.21; *SD* = 0.67) and denial (mean = 2.66; *SD* = 1.09).

Depression (mean = 4.77; *SD* = 5.78), anxiety (mean = 3.20; *SD* = 4.67), and stress (mean = 8.01; *SD* = 7.02) scores were in the normal range compared to the scores described in the validated questionnaire (20). Regarding the perception of illness (BIPQ), the mean score was slightly above the average in the normal range in the validated questionnaire (mean = 42.09; *SD* = 9.26). On the other hand, the workers referred to good social support (EMAS) in the global score (mean = 70.53; *SD* = 15.06), as well as in each dimension of this scale (friends, family, and relevant persons) (Table 1).

### 3.2. Factors Related to Depression, Anxiety, and Stress in the Study Population: Bivariate Analyses

As Table 2 shows, women had higher average scores than men for depression (5.56 vs. 3.96 in men), anxiety (3.95 vs. 2.45 in men), and stress (9.46 vs. 6.55 in men), while the scores in the ASP group were higher than those in the RTP group on the depression and anxiety scale (5.45 vs. 4.35 in RTP and 3.54 vs. 3.00 in RTP, respectively). Moreover, the mean scores for depression decreased as the subject level of education increased (8.0 in primary studies, 5.5 in secondary, and 4.67 in university studies). In addition, the scores for depression, anxiety, and stress were higher among the subjects living in a smaller home, and if their home had no open areas. Age was inversely related to the scores for depression (Spearman’s rank correlation coefficient (Rho) = −0.106), anxiety (Rho = −0.117), and stress (Rho = −0.169) (Table 2).

The people whose health status was worse or much worse than before the lockdown reported higher levels of depression, anxiety, and stress. Likewise, these levels were higher in people with a personal history of chronic illness (mean depression = 6.06 vs. 4.12; mean anxiety = 4.55 vs. 2.53; mean stress = 9.55 vs. 7.25) and among those suffering from chronic pain (mean anxiety = 4.53 vs. 2.98; mean stress = 10.43 vs. 7.62). The university workers with worse results for the sleep-related questions had higher levels of depression, anxiety, and stress (Table 2).

Depression increased when some positive coping strategies decreased (emotion-focused or problem-focused strategies, such as active coping (Rho = −0.108), positive reframing (Rho = −0.111), and acceptance (Rho = −0.270)), and increased when emotional support (Rho = 0.146), instrumental support (Rho = 0.127), religion (Rho = 0.151), and all dysfunctional coping strategies increased too. On the other hand, anxiety and stress scores decreased with acceptance (Rho = −0.193 and Rho = −0.208, respectively) and increased with emotional support (Rho = 0.132 and Rho = 0.202), instrumental support (Rho = 0.141 and Rho = 0.204), religion (Rho = 0.159 and Rho = 0.117), and dysfunctional coping strategies (Table 2).

Depression, anxiety, and stress levels increased as the BIPQ score increased. Moreover, when social support decreased, depression (Rho = −0.169), anxiety (Rho = −0.115), and stress (Rho = −0.108) increased. A similar situation was observed for its three dimensions (Table 2).

### 3.3. Factors Related to Depression, Anxiety, and Stress in the Study Population: Multivariate Analyses

The results found using the linear regression models for the level of depression, anxiety, and stress are shown in Table 3, Table 4 and Table 5, respectively.

Having more problems sleeping at night and some coping strategies such as denial (Beta coefficient (*B)* = 0.498), self-distraction (*B* = 0.292), self-blame (*B* = 0.489), and venting (*B* = 0.258) were related to higher levels of depression. Additionally, higher levels of depression were observed in people with higher levels of anxiety (*B* = 2.692), stress (*B* = 1.735), worse illness perception (*B* = 0.047), and a history of chronic disease (*B* = 0.828). In contrast, the levels of depression decreased by 0.046 points for each year of age, in people sleeping nine or more hours (*B* = −2.019) and with greater self-perceived family social support (*B* = −0.153) (Table 3).

Regarding anxiety, we found that the score increased in people with a history of chronic disease (*B* = 0.695) and a worse illness perception (*B* = 0.034). Only one coping strategy (humour) was related to higher anxiety scores (*B* = 0.155). The scores for depression (*B* = 0.348) and stress (*B* = 0.247) were also directly related to the score for anxiety. Meanwhile, living in a bigger home was associated with lower anxiety scores (Table 4).

As for the factors associated with the stress, we found that people with a history of chronic disease had higher scores of stress (*B* = 1.197), a phenomenon also observed in people who sometimes (*B* = 1.748) or usually (*B* = 2.421) had trouble falling asleep, and in those that usually dreamt of what was happening (*B* = 1.795). Two coping strategies were related to higher scores for stress: planning (*B* = 0.423) and venting (*B* = 0.616). Again, higher scores for depression (*B* = 0.408), anxiety (*B* = 0.526) and worse illness perception (*B* = 0.090) were related to higher stress scores. On the other hand, the stress score decreased as age increased (*B* = −0.044), and when the denial strategy was used (*B* = −0.418) (Table 5).

## 4. Discussion

This study analyses the impact of the COVID-19-related lockdown on the levels of depression, anxiety, and stress in a group of university workers, in addition to the factors related to these disorders. The results obtained indicate that even at the beginning of the pandemic, the lockdown affected these workers to a certain extent. The highest scores for depression, anxiety, and stress were obtained by women, younger subjects, administration and service workers (ASP sector), and people with a lower educational level, as well as those with a worse health status, worse illness perception, worse quality of sleep, or using dysfunctional coping strategies. Furthermore, other social factors, such as living in a smaller home, had a negative impact on the psychological processes analysed, while more family support was related to lower depression scores.

The greater impact of the lockdown on the depression, anxiety, and stress scores observed among women is in line with the findings of Wang et al. [20], whereby women have higher levels of these variables within this context. Likewise, Li et al. [36] argue that women are more likely to have anxiety and depression symptoms during the spread of an epidemic, possibly because they are more sensitive to personal growth and interpersonal relationships than men, as previously shown in general population [37].

Regarding age, other studies have shown similar results to ours, with younger subjects presenting higher scores for depression, anxiety, and stress [38,39]. It is possible that, in addition to the effects deriving from difficulty socializing during lockdown, young people suffer from a worse emotional state as a result of having less stable jobs and being more concerned about their future career. Accordingly, a survey conducted in the United States at the beginning of the pandemic found that being young was associated with a higher presence of anxiety and depression, and being more worried about experiencing economic hardship [40]. The present study found that the mean age of the workers with temporary contracts ranged between 30 and 40, while those with permanent contracts had a mean age over 49 (*p* < 0.001, data not shown). Consistently, the mean age was lower among the subjects that were more worried about losing their job (45 years vs. 52; *p* < 0.001, data not shown).

Furthermore, this study found higher depression and anxiety scores in the ASP group than among the research and teaching personnel (RTP). In addition, the mean depression scores decreased as the subjects’ level of education increased. These two variables (sector and study level) are related, as all the RTP in the sample had university studies, while 27.3% of the ASP had primary or secondary studies (*p* < 0.001, data not shown). However, other authors, such as Wang et al. [20], found no relationship between depression, anxiety, and stress scores and educational level. This suggests that the differences observed in this study are connected more with the participants’ sector of work than their level of studies. In this sense, a possible explanation could be the fact that, unlike the RTP, it was not easy for the ASP to adapt to working online, which could have led to a worse emotional state.

The participants’ perception that their health state had worsened during lockdown is also related to higher depression, anxiety, and stress scores. Similar results have been found by other authors [41], who argue that people who perceive their health status to be worse feel that they are more likely to be infected and less likely to survive, which would have a negative impact on their emotional state.

The present study observed that worse sleep characteristics were related to higher depression, anxiety, and stress scores. In this regard, other authors [42,43] have reported that people that had trouble getting to sleep or staying asleep, or that thought about stressful events, had a greater risk of depression [43]. Likewise, experiences in life itself, especially stressful events, have been said to affect sleep quality, possible influencing sleep patterns and content [44]. It is worth noting that the relationship between depression, anxiety, and sleep disorders has been described in several pathological processes, which suggests that common neurobiological mechanisms may exist that could result in a feedback loop [45].

Regarding the coping strategies developed by the participants of the study, it is of note that active coping was associated with lower scores on the depression scale, these results being in line with those of Torres et al. [46]. Likewise, emotion-focused strategies, such as accepting that what is happening is real or the use of humour, were also associated with lower scores on this scale. McWilliams et al. [47] also showed that emotion-focused strategies are associated with a decrease in depression.

In the case of anxiety, the only related coping strategy was the use of humour, which was associated with higher anxiety scores. This result appears, a priori, to contradict the fact that humour is associated with lower levels of depression, although it could be justified by the different interpretations of this sub-scale in the literature. Besser et al. [48] found that maladaptive humour—that which is used to gain approval or for ridiculing others or oneself—could increase levels of anxiety. However, although this theoretical framework exists, our data does not provide any guarantee that the humour was maladaptive in this context.

One of the coping strategies associated with a higher level of stress was venting, which has been reported previously by Gurvich et al. [49]. This makes sense, because venting has been classified as a dysfunctional strategy and was considered less useful in earlier coping models, such as the one by Stanislawski [50]. However, a greater use of planning, considered to be an appropriate coping strategy, was also associated with higher levels on this scale. The study by Umucu and Lee [51], conducted during the COVID-19 pandemic in patients with chronic pathologies, reported the same relationship and in fact, found higher levels of stress when both adaptive and maladaptive strategies were used. For this reason, the authors deliberate the need to reconsider our understanding of the use of coping strategies, at least as far as their impact on stress levels is concerned. Finally, denial was associated with lower scores on the stress scale. An explanation for this could be what Stanislawski referred to as “hedonistic disengagement” [50], which involves avoiding and denying information about the problem and a strong tendency to maintain momentary well-being, thereby possibly reducing stress levels.

As observed in this study, other social variables, such as having a smaller home, have been highlighted in the literature as factors that can lead to a greater risk of anxiety [24]. Small apartments with little natural light and few living spaces may be insufficient to provide the occupants with privacy, and may make relationships between them more difficult. Furthermore, the study by Amerio et al. [24] in a university in northern Italy concluded that workers having to live in a small area without a workplace available that makes it possible to differentiate work and leisure time may be less productive, and consequently, suffer from increased anxiety.

Finally, our study also showed that the perception of less social support from the family was also related to higher depression scores, as other authors have reported [34,52]. In particular, Hoffart et al. [52] showed that loneliness due to the COVID-19-related lockdown was a potential risk factor for increases in depressive symptoms.

## 5. Limitations and Strengths

Some limitations should be considered in the study. First, voluntary participation in the survey, and the fact that people with more limitations or worse physical or mental health might not have answered the survey, could have biased the sample. Indeed, in the study, the percentage of respondents with moderate to severe depression, anxiety, or stress was around 7% (data not shown), lower than those found by Odriozola et al. [13] in another university. However, in this study, 76% of the sample consisted of students, who showed higher scores on the DASS-21 scale, which could justify the differences observed with our results. In addition, in order to rule out the possibility of bias related to voluntary participation, we compared the sample with the total population in terms of the distributions by gender and sector, the two groups being virtually the same. This indicates that there is no evidence to suspect a possible bias in the sample. In spite of this, the findings cannot be extrapolated to the general population, because the study was carried out in a university worker population in a regional area. Also, the cross-sectional nature of the study does not make it possible to establish causal relationships between the variables. However, this is an initial approach to clarify these relationships, and further longitudinal studies are needed to check them.

A potential bias that could affect the results obtained is that the participants were not asked about the previous history of anxiety, depression, or stress. However, we believe that the use of scales with adequate psychometric properties provides accurate and adequate information for the cross-sectional design of the study. Another aspect to bear in mind is the possible influence of the presence of friends and family members affected by COVID-19 in the responses of the participants. In this respect, we found that 17.4% of the respondents had a friend or relative with positive results to COVID-19, but only one person was actually living with the affected friend or relative (data not shown).

As for strengths, we have to point out the limited information on the consequences of the lockdown in university workers, the use of validated scales, and a large sample size.

## 6. Conclusions

The COVID-19-related lockdown had a great impact on university workers. The subjects with specific sociodemographic and occupational characteristics, clinical disorders, and dysfunctional coping strategies were more at risk for depression, anxiety, and stress.

The emotional effect of the lockdown makes it necessary to assess and monitor the health of university workers from a biopsychosocial perspective, as well as the inclusion of multidimensional strategies that improve the quality of life of the population and prevent future mental and physical problems derived from this and future pandemics.

A year from when the COVID-19 pandemic began, it would be of interest to perform new studies to identify the possible consequences of the disease and social isolation in this group.

## Figures and Tables

**Table 1:** Characteristics of the sample.

Variable	Category	*n* (%)
Gender (*n* = 676)	Men	337 (49.90%)
	Women	339 (50.10%)
Age (*n* = 666)	Mean (*SD*)	48.75 (10.51%)
Marital status (*n* = 676)	Married or in a couple	491 (72.60%)
	Divorced or separated	52 (7.70%)
	Widow(er)	7 (1.00%)
	Single	126 (18.60%)
Study level (*n* = 674)	Primary	3 (0.40%)
	Secondary	66 (9.80%)
	University	605 (89.80%)
Sector (*n* = 677)	RTP	423 (62.50%)
	ASP	254 (37.50%)
Children under 14 years old living with you (*n* = 669)	0	490 (73.50%)
	1	89 (13.30%)
	2	72 (10.80%)
	3 or more	16 (2.30%)
People dependent on your care, over 14 years old (*n* = 659)	0	525 (79.70%)
	1–2	121 (18.50%)
	3 or more	13 (2.00%)
Size of your home (*n* = 674)	Up to 49 m^2^	20 (3.0%)
	50–89 m^2^	242 (35.90%)
	90–129 m^2^	250 (37.10%)
	130 m^2^ or more	162 (24.00%)
Does your home have open areas like a garden, terrace, patio, or porch? (*n* = 674)	No	279 (41.40%)
	Yes	395 (58.60%)
Current health status compared to health status before the lockdown	Much worse	6 (0.90%)
	Worse	96 (14.20%)
	Same	545 (80.50%)
	Better	30 (4.40%)
	Much better	0 (0.0%)
Personal history of chronic disease	Yes	224 (33.10%)
	No	453 (66.90%)
Chronic pain	Yes	94 (13.90%)
	No	583 (86.10%)
Sleep quality during the state of alarm (*n* = 675)	Very good	79 (11.70%)
	Good	229 (33.90%)
	Not Good, not bad	210 (31.10%)
	Bad	134 (19.90%)
	Very bad	23 (3.40%)
Number of hours per day of sleep	Less than 6	37 (5.50%)
	6–8	597 (88.20%)
	9 or more	43 (6.40%)
Trouble sleeping at night	Never	150 (22.20%)
	Hardly ever	196 (29.00%)
	Sometimes	195 (28.80%)
	Usually	136 (20.10%)
Trouble falling asleep	Never	163 (24.10%)
	Hardly ever	224 (33.10%)
	Sometimes	179 (26.40%)
	Usually	111 (16.40%)
Dreams about what is happening	Never	369 (54.50%)
	Hardly ever	147 (21.70%)
	Sometimes	124 (18.30%)
	Usually	37 (5.50%)
**Scale**	**Range**	**Mean (*SD*)**
Brief-COPE dimensions: emotion-focused strategies	10–40	23.57 (4.58)
Emotional support	2–8	4.48 (1.57)
Positive reframing	2–8	5.17 (1.56)
Acceptance	2–8	6.46 (1.22)
Religion	2–8	3.23 (1.66)
Humour	2–8	4.22 (1.69)
Brief-COPE dimensions: problem-focused strategies	6–24	15.08 (3.27)
Active coping	2–8	5.63 (1.36)
Planning	2–8	5.39 (1.46)
Instrumental support	2–8	4.05 (1.30)
Brief-COPE dimensions: dysfunctional coping strategies	12–48	19.73 (3.51)
Denial	2–8	2.66 (1.09)
Self-distraction	2–8	5.09 (1.42)
Self-blame	2–8	3.26 (1.13)
Behavioural disengagement	2–8	2.71 (1.12)
Venting	2–8	3.78 (1.28)
Substance use	2–8	2.21 (0.67)
DASS-21 dimensions: depression	0–42	4.77 (5.78)
DASS-21 dimensions: anxiety	0–42	3.20 (4.67)
DASS-21 dimensions: stress	0–42	8.01 (7.02)
BIPQ Illness perception	0–80	42.09 (9.26)
EMAS social support (total)	12–84	70.53 (15.06)
Dimensions: friends social support	4–28	22.60 (5.74)
Dimensions: family social support	4–28	23.90 (5.33)
Dimensions: relevant person social support	4–28	24.03 (5.70)

*SD*: standard deviation; RTP: research and teaching personnel; ASP: administration and services personnel; DASS: Depression Anxiety Stress Scales; BIPQ: Brief Illness Perception Questionnaire; EMAS: Escala Multidimensional de Apoyo Social [Social Support Multidimensional Scale].

**Table 2:** Factors related to the scores for depression, anxiety, and stress: bivariate analysis.

Variable	Category	Depression Mean (*SD*)	*p* ^1^	Anxiety Mean (*SD*)	*p* ^2^	Stress Mean (*SD*)	*p* ^3^
Gender (*n* = 676)	Men	3.96 (4.95)	<0.001 ^a^	2.45 (3.99)	<0.001 ^a^	6.55 (6.42)	<0.001 ^a^
	Women	5.56 (6.41)		3.95 (5.16)		9.46 (7.32)	
Age (*n* = 666)		Rho = −0.106	0.006 ^b^	Rho = −0.117	0.003 ^b^	Rho = −0.169	<0.001^b^
Marital status (*n* = 676)	Married or in couple	4.37 (5.23)	0.133 ^c^	2.98 (4.465)	0.388 ^c^	7.78 (6.83)	0.582 ^c^
	Divorced or separated	4.92 (4.16)		3.69 (4.16)		5.58 (5.76)	
	Single	6.25 (7.91)		3.90 (5.62)		8.68 (8.21)	
	Widow(er)	4.00 (3.83)		2.29 (2.14)		7.14 (6.20)	
Sector (*n* = 677)	RTP	4.35 (5.55)	0.004 ^a^	3 (4.73)	0.031 ^a^	7.76 (7.15)	0.095 ^a^
	ASP	5.45 (6.09)		3.54 (4.56)		8.43 (6.80)	
Education level (*n* = 674)	Primary	8.00 (0)	0.031 ^c^	4.67 (2.31)	0.274 ^c^	8.67 (3.06)	0.776 ^c^
	Secondary	5.55 (5.33)		3.58 (4.46)		7.45 (6.59)	
	University	4.67 (5.84)		3.15 (4.71)		8.07 (7.09)	
Children under 14 years old living with you (*n* = 667)	0	4.89 (5.93)	0.667 ^c^	3.30 (4.69)	0.886 ^c^	7.91 (7.10)	0.394 ^c^
	1	4.31 (5.14)		2.79 (3.77)		8.09 (6.86)	
	2	4.67 (5.45)		3.00 (5.08)		8.53 (6.96)	
	3 or more	6.00 (6.69)		4.13 (7.13)		10.25 (7.00)	
People over 14 dependent on your care (*n* = 659)	0	4.84 (6.04)	0.327 ^c^	3.28 (4.79)	0.395 ^c^	8.10 (7.31)	0.389 ^c^
	1–2	4.79 (4.90)		3.21 (4.50)		8.17 (6.11)	
	3 or more	3.08 (3.97)		1.85 (3.31)		5.54 (4.91)	
Size of your home (*n* = 674)	Up to 49 m^2^	7.90 (9.410)	<0.001 ^c^	6.30 (6.46)	0.001 ^c^	10.20 (7.68)	0.008 ^c^
	50–89 m^2^	5.69 (6.480)		3.82 (5.25)		8.98 (7.19)	
	90–129 m^2^	4.34 (4.920)		2.74 (4.01)		7.51 (6.94)	
	130 m^2^ or more	3.68 (5.014)		2.63 (4.24)		7.06 (6.70)	
Home open areas like garden, terrace, patio, or porch (*n* = 674)	No	5.30 (6.28)	0.046 ^a^	3.68 (5.13)	0.017 ^a^	8.61 (7.13)	0.075 ^a^
	Yes	4.39 (5.39)		2.87 (4.30)		7.58 (6.82)	
Current health status compared to health status before the lockdown	Much worse	7.69 (7.18)	<0.001 ^c^	6.48 (6.52)	<0.001 ^c^	12.67 (8.17)	<0.001^c^
	Worse	6.00 (6.57)		6.33 (6.62)		9.33 (5.47)	
	Same	4.25 (5.36)		2.62 (4.05)		7.18 (6.57)	
	Better	4.53 (5.48)		2.67 (3.12)		7.87 (5.91)	
Personal history of chronic illness	No	4.12 (5.03)	<0.001 ^a^	2.53 (3.84)	<0.001 ^a^	7.25 (6.70)	<0.001 ^a^
	Yes	6.06 (6.88)		4.55 (5.78)		9.55 (7.42)	
Suffer from chronic pain	No	4.63 (5.65)	0.182 ^a^	2.98 (4.39)	0.015 ^a^	7.62 (6.67)	0.005 ^a^
	Yes	5.60 (6.44)		4.53 (5.98)		10.43 (8.57)	
Sleep quality (*n* = 675)	Very bad	7.57 (7.08)	<0.001 ^c^	5.83 (6.71)	<0.001 ^c^	12.78 (8.24)	<0.001 ^c^
	Bad	7.91 (6.86)		5.94 (6.14)		12.27 (7.52)	
	Not good, not bad	5.05 (5.61)		3.22 (4.31)		8.55 (6.68)	
	Good	3.46 (4.84)		2.11 (3.52)		6.22 (6.01)	
	Very good	1.62 (2.64)		0.94 (1.46)		3.24 (3.90)	
Number of hours per day of sleep	Less than 6	7.95 (8.45)	0.012 ^c^	5.73 (7.86)	0.052 ^c^	12.27 (7.71)	0.001 ^c^
	6–8	4.60 (5.54)		3.03 (4.36)		7.78 (6.83)	
	9 or more	4.28 (5.44)		3.44 (4.75)		7.58 (8.03)	
Trouble sleeping at night	Never	2.23 (3.20)	<0.001 ^c^	1.43 (2.30)	<0.001 ^c^	4.65 (5.75)	<0.001 ^c^
	Hardly ever	4.27 (5.96)		2.55 (4.05)		7.28 (6.53)	
	Sometimes	5.44 (5.50)		3.52 (4.50)		8.86 (6.53)	
	Usually	7.32 (6.80)		5.63 (6.35)		11.54 (7.79)	
Trouble falling asleep	Never	2.12 (3.98)	<0.001 ^c^	1.18 (2.22)	<0.001 ^c^	4.13 (4.93)	<0.001 ^c^
	Hardly ever	4.47 (5.30)		2.72 (3.86)		7.42 (5.98)	
	Sometimes	5.58 (5.85)		4.11 (5.03)		9.47 (7.18)	
	Usually	7.93 (6.90)		5.66 (6.46)		12.52 (8.04)	
Dreams about what is happening	Never	3.31 (4.71)	<0.001 ^c^	2.08 (3.31)	<0.001 ^c^	5.97 (5.91)	<0.001 ^c^
	Hardly ever	4.94 (4.68)		3.05 (3.75)		8.56 (5.74)	
	Sometimes	7.35 (7.14)		5.29 (6.33)		11.10 (8.26)	
	Usually	9.89 (8.06)		8.00 (7.20)		15.78 (8.06)	
**Scale**		**Rho**	***p*** **^1^**	**Rho**	***p*** **^2^**	**Rho**	***p*** **^3^**
Emotion-focused strategies		−0.052	0.179 ^b^	0.014	0.718 ^b^	0.027	0.481 ^b^
Emotional support		0.146	<0.001 ^b^	0.132	0.001 ^b^	0.202	<0.001 ^b^
Positive reframing		−0.111	0.004 ^b^	−0.063	0.101 ^b^	−0.039	0.314 ^b^
Acceptance		−0.270	<0.001 ^b^	−0.193	<0.001 ^b^	−0.208	<0.001 ^b^
Religion		0.151	<0.001 ^b^	0.159	<0.001 ^b^	0.117	0.002 ^b^
Humor		−0.064	0.096 ^b^	0.026	0.505 ^b^	−0.005	0.906 ^b^
Problem-focused strategies		−0.044	0.252 ^b^	0.051	0.187 ^b^	0.130	0.001 ^b^
Active coping		−0.108	0.005 ^b^	−0.004	0.815 ^b^	0.051	0.183 ^b^
Planning		−0.103	0.007 ^b^	−0.004	0.907 ^b^	0.061	0.114 ^b^
Instrumental support		0.127	0.001 ^b^	0.141	<0.001 ^b^	0.204	<0.001 ^b^
Dysfunctional coping strategies		0.373	<0.001 ^b^	0.332	<0.001 ^b^	0.386	<0.001 ^b^
Denial		0.268	<0.001 ^b^	0.244	<0.001 ^b^	0.179	<0.001 ^b^
Self-distraction		0.229	<0.001 ^b^	0.200	<0.001 ^b^	0.260	<0.001 ^b^
Self-blame		0.167	<0.001 ^b^	0.166	<0.001 ^b^	0.219	<0.001 ^b^
Behavioral disengagement		0.163	<0.001 ^b^	0.124	0.001 ^b^	0.108	0.005 ^b^
Venting		0.214	<0.001 ^b^	0.194	<0.001 ^b^	0.278	<0.001 ^b^
Substance use		0.156	<0.001 ^b^	0.138	<0.001 ^b^	0.157	<0.001 ^b^
BIPQ		0.403	<0.001 ^b^	0.412	<0.001 ^b^	0.432	<0.001 ^b^
Social support		−0.169	<0.001 ^b^	−0.115	0.003 ^b^	−0.108	0.005 ^b^
Friends social support		−0.149	<0.001 ^b^	−0.118	0.002 ^b^	−0.095	0.014 ^b^
Family social support		−0.199	<0.001 ^b^	−0.114	0.003 ^b^	−0.134	<0.001 ^b^
Relevant person social support		−0.128	0.001 ^b^	−0.078	0.043 ^b^	−0.070	0.068 ^b^

*SD*: standard deviation; RTP: research and teaching personnel; ASP: administration and services personnel; Rho: Spearman’s rank correlation coefficient; BIPQ: Brief Illness Perception Questionnaire; *p*^1^: *p*-value for depression, *p*^2^: *p*-value for anxiety, *p*^3^: *p*-value for stress; ^a^ Mann–Whitney U; ^b^ Spearman rank correlation coefficient; ^c^ Kruskal–Wallis H.

**Table 3:** Factors related to depression scores. Linear regression model.

Variable	Category/Unit	B	SE (B)	*p*	95% CI	
Age	Years	−0.046	0.016	0.003	−0.077	−0.016
History of chronic disease (Ref.: no)	Yes	0.828	0.334	0.014	0.172	1.484
Number of hours per day of sleep (Ref.: less than 6)	6–8 hours	−0.797	0.694	0.251	−2.161	0.566
	9 or more hours	−2.019	0.908	0.027	−3.801	−0.236
Trouble sleeping at night (Ref.: never)	Hardly ever	0.951	0.426	0.026	0.114	1.789
	Sometimes	1.126	0.437	0.010	0.268	1.983
	Usually	1.293	0.504	0.011	0.303	2.283
Brief-COPE28 active coping	Scale 2–8	−0.330	0.128	0.010	−0.582	−0.079
Brief-COPE28 acceptance	Scale 2–8	−0.439	0.140	0.002	−0.714	−0.165
Brief-COPE28 denial	Scale 2–8	0.498	0.150	0.001	0.204	0.792
Brief-COPE28 humour	Scale 2–8	−0.237	0.100	0.019	−0.433	−0.040
Brief-COPE28 self-distraction	Scale 2–8	0.292	0.114	0.011	0.068	0.517
Brief-COPE28 self-blame	Scale 2–8	0.489	0.150	0.001	0.195	0.783
Brief-COPE28 venting	Scale 2–8	0.258	0.130	0.048	0.003	0.513
DASS-21 level of anxiety	Scale 0–42	2.692	0.267	<0.001	2.168	3.217
DASS-21 level of stress	Scale 0–42	1.735	0.305	<0.001	1.137	2.333
BIPQ illness perception	Scale 0–80	0.047	0.019	0.011	0.011	0.084
EMAS: family social support	Scale 4–28	−0.153	0.029	<0.001	−0.211	−0.096

B: beta coefficient; SE: standard error; *p*: *p*-value; CI: confidence interval; *R*^2^ = 0.569.

**Table 4:** Factors related to anxiety scores: linear regression model.

Variable	Category/Unit	B	SE (B)	*p*	95% CI	
Size of your home	50–89 m^2^	−1.483	0.664	0.026	−2.786	−0.179
	90–129 m^2^	−1.666	0.665	0.013	−2.972	−0.359
	130 m^2^ or more	−1.389	0.680	0.042	−2.724	−0.053
History of chronic disease (ref: no)	Yes	0.695	0.237	0.003	0.230	1.161
Brief-COPE28: humour	Scale 2–8	0.155	0.066	0.019	0.025	0.284
DASS-21 level of depression	Scale 0–42	0.348	0.027	0.000	0.295	0.402
DASS-21 level of stress	Scale 0–42	0.247	0.023	0.000	0.202	0.292
BIPQ illness perception	Scale 0–80	0.034	0.013	0.012	0.008	0.060

B: beta; SE: standard error; *p*: *p*-value; CI: confidence interval; *R*^2^ = 0.635.

**Table 5:** Factors related to stress scores. Linear regression model.

Variable	Category/Unit	B	SE (B)	*p*	95% CI	
Age	Years	−0.044	0.016	0.006	−0.075	−0.012
Chronic pain (ref.: no)	Yes	1.197	0.479	0.013	0.257	2.138
Trouble falling asleep (ref.: never)	Hardly ever	1.095	0.440	0.013	0.230	1.959
	Sometimes	1.748	0.486	0.000	0.793	2.702
	Usually	2.421	0.566	0.000	1.310	3.532
Dreams about what is happening (ref.: never)	Hardly ever	0.860	0.419	0.040	0.037	1.682
	Sometimes	0.768	0.473	0.105	−0.160	1.697
	Usually	1.795	0.792	0.024	0.239	3.350
Brief-COPE strategies: planning	Scale 2–8	0.423	0.118	0.000	0.191	0.655
Brief-COPE28 denial	Scale 2–8	−0.418	0.160	0.009	−0.732	−0.103
Brief-COPE28 venting	Scale 2–8	0.616	0.135	0.000	0.351	0.882
DASS-21 level of depression	Scale 0–42	0.408	0.042	0.000	0.325	0.491
DASS-21 level of anxiety	Scale 0–42	0.526	0.053	0.000	0.422	0.630
BIPQ illness perception	Scale 0–80	0.090	0.020	0.000	0.051	0.129

B: beta; SE: standard error; *p*: *p*-value; CI: confidence interval; *R*^2^ = 0.658.

## Data Availability

The data presented in this study are available on request from the corresponding author. The data are not publicly available due to privacy.

## Supplementary Materials

The following are available online at https://www.mdpi.com/article/10.3390/ijerph18084367/s1, Table S1: Brief COPE 28 internal structure.
