# Supplementary material for: Psychological Impact of the Lockdown Due to the COVID-19 Pandemic in University Workers: Factors Related to Stress, Anxiety, and Depression
Source: Int J Environ Res Public Health. 2021 Apr 20;18(8):4367. doi: 10.3390/ijerph18084367 (PMC8074294; doi:10.3390/ijerph18084367)
Supplement: Supplementary file 1 [file ijerph-18-04367-s001.zip › ijerph-1155900-supplementary.pdf]

Table S1. Brief COPE 28 internal structure.

Dimension	Subscale	Subscale definition
Dimension 1, Emotion-focused strategies	4. Emotional support	Getting emotional support/comfort and understanding.
	8. Positive reframing	Trying to see it in a different light, make it seem more positive/ look for something good in it.
	10. Acceptance	Accepting the reality that it has happened/ learning to live with it.
	11. Religion	Finding comfort in religious or spiritual beliefs/ praying or meditating.
	13. Humor	Making jokes about the stressor/making fun on the situation.
Dimension 2, Problem-focused strategies	1. Active coping	Concentrating my efforts on doing something about the situation I'm in/ taking action to try to make it better
	2. Planning	Trying to come up with a strategy about what to do/ thinking hard about what steps to take.
	3. Instrumental support	Getting help and advice from other people/ trying to get advice or help from others about what to do.
Dimension 3, Dysfunctional coping strategies	5. Self-distraction	Turning to work or other activities to take my mind off things/ doing something to think about it less.
	6. Venting	Saying things to let unpleasant feelings escape/ expressing negative feelings
	7. Behavioral disengagementl	giving up trying to deal with it/ the attempt to cope.
	9. Denial	Saying to myself "this isn't real" /refusing to believe that it has happened.
	12. Substance use	Using alcohol or other drugs to make myself feel better/ to help me get through it
	14. Self-blame	Criticizing myself/ blaming myself for things that happened.

Source: Cooper, C.; Katona, C.; Livingston, G. Validity and reliability of the brief cope in carers of people with dementia: 333 The LASER-AD study. J. Nerv. Ment. Dis. 2008, 196, 838–843, doi:10.1097/NMD.0b013e31818b504c.
